# Patriotism of the Nurse-Training Schools

**Published:** 1916-12-30

**Authors:** 


### PATRIOTISM OF THE NURSE-TRAINING SCHOOLS.

### XI. War Work in Glasgow Institutions.

Nothing is more interesting than to trace the sacrifices made in special towns for the cause of the wounded. Our readers will agree that the succeeding paragraphs give a fine illustration of what Glasgow has done and is doing in the war.

### Royal Infirmary, Glasgow.

Since the war began about ninety members of the nursing staff have been engaged in war work. Of these, thirty-eight went to Territorial hospitals at home, and many have since been drafted abroad. Twenty-five members from the Civil Hospital List went to the Q.A.I.M.N.S.R.; seventeen to the Q.A.N.N.S.R., and twenty-five to the Territorial Force Nursing Service. In addition, there are at least sixty nurses in the Services who were trained at the Royal Infirmary, some being with the Red Cross Society, four with the French Flag Nursing Corps, two with the Serbian Unit, and three with the Scottish Women's Hospital. The matron, Miss Melrose, is also principal matron of the 4th Scottish General Hospital, Stobhill, and Merryflatts War Hospital. Three of her assistants are engaged in war work, one is the matron of the 4th Scottish General Hospital, and two are in France. Four of the present staff at No. 4 Scottish General Hospital have received the Royal Red Cross, and many have been mentioned in dispatches. Ten of those trained in the Royal Infirmary have also been mentioned in dispatches, and six have received the Royal Red Cross.

### The Western Infirmary of Glasgow.

Few hospitals have given more abundantly of their best than the "Western." The night superintendent, Miss A. Matheson, is now sister in the Q.A.I.M.N.S.R.; the second assistant matron, Miss A. H. Kerr, and the home sister, Miss R. J. G. Steel, are both matrons in the Territorial Force Nursing Service. No fewer than thirteen ward sisters have left the infirmary to take posts in the Q.A.R.N.N.S., Q.A.I.M.N.S.R. and in the T.F.N.S. Their names are: Sisters L. L. Brown, M. G. Campbell, A. Cunningham, I. Baillie, A. M. Lockhart, M. D. Bain, A. Stevenson, J. C. Kirkpatrick, J. Henry, M. M. Tait, J. W. Angus, G. Sanderson, and Mrs. E. M. Hannay (Kunhardt). The following ten staff nurses were called up on Q.A.R.N.N.S.R. and Q.A.I.M.N.S.R.:—Nurses M. Cunningham, B. Allardes, K. W. White, E. D. Altham, J. K. Lockie, F. Laidler, M. J. Peebles, E. Robertson, G. Davidson, and K. Love. In addition to those already named, thirty-four nurses who left on the completion of their training are doing war nursing, and of these the following twelve have been appointed staff nurses in the Territorial Force Nursing Service: Nurses E. T. Wilson, M. Baxter, E. Macleod, M. I. Mitchell, B. F. Duthie, N. R. Farquharson, I. Henderson, E. A. Clark, C. D. Spinks, J. M. Clark, K. Macleay, and H. H. Archibald. A very large number of sisters and nurses who received their training at the Western and who had left before the outbreak of the war are doing good work for the wounded in various parts of the world. The matron is indebted to many of the late members, several of whom are married, for loyally returning to relieve the sisters called up for duty in the various Services. The following sisters have been mentioned in dispatches—namely: Miss Margaret D. Bain (ward-sister), sister, T.F.N.S.; Miss Elizabeth Kerr (late ward-sister), sister, T.F.N.S.; and the following five members have been awarded the decoration of the Royal Red Cross—namely: Miss Alicia Hope Kerr (second assistant matron), matron, T.F.N.S. (First Class); Miss Sybil Edgar (ward sister), Second Class; Miss Lucy M'Lean (late assistant matron), Second Class; Miss Catherine M'Callum (trained Western Infirmary), sister, T.F.N.S. (Second Class); Miss Mary Donald (trained Western Infirmary), staff-nurse, T.F.N.S. (Second Class).

### Victoria Infirmary, Glasgow.

Miss Rodger, the assistant matron, is with the British Expeditionary Force in France. Sisters I. Calder, E. Neil, H. Morrice, S. Cowell, M. Smith, and A. McCall are nursing in military hospitals in Glasgow and elsewhere. Sister Charlotte Smith is in the Plymouth Naval Hospital. Sister M. Houston, after nursing under the Red Cross in Serbia, has returned to the infirmary. Nurses E. Nicholson, A. Wotherspoon, M. Connell, A. Macdonald (since resigned), and L. Crawford went to military hospitals at home, and Nurse C. Shanks is at Haslar Naval Hospital. Thus eight sisters out of thirteen have been surrendered to war work. The following nurses, trained at the infirmary, have gone to take up war duty since the beginning of this year:—Nurses J. Smith and M. Bridson to France; Nurses I. Cameron, H. Douglas, L. Allan, I. Walker, M. Bell, M. Morgan, M. McKnight, C. Macnaughton to military hospitals in the United Kingdom.

### Western Glasgow District Hospital, Oakbank.

This infirmary, containing 250 beds, is in normal times a good training school, and is recognised by the Central Midwives Board. On June 1, 1915, it was taken over, with the entire staff, by the military authorities. No new probationers are being received, but the training of those already there when the hospital was taken over is continuing as before, and the trained staff connected with the institution are now all members of the T.F.N.S. The ordinary patients have been transferred elsewhere.

### Royal Alexandra Infirmary, Paisley.

The following members of the staff have left on war service: Miss Yule (assistant matron), and Nurses Rankine and Mair, working in military hospital in Rouen; Sister Mary Brown and Nurse Macfarlane, on hospital ships.

